# Eight-hours conventional versus adaptive deep brain stimulation of the subthalamic nucleus in Parkinson’s disease

**DOI:** 10.1038/s41531-021-00229-z

**Published:** 2021-09-28

**Authors:** Tommaso Bocci, Marco Prenassi, Mattia Arlotti, Filippo Maria Cogiamanian, Linda Borellini, Elena Moro, Andres M. Lozano, Jens Volkmann, Sergio Barbieri, Alberto Priori, Sara Marceglia

**Affiliations:** 1 “Aldo Ravelli” Research Center for Neurotechnology and Experimental Brain Therapeutics, Department of Health Sciences, University of Milan Medical School, Milan, Italy; 2 III Neurology Clinic, ASST Santi Paolo e Carlo, Milan, Italy; 3 Dipartimento in Ingegneria e Architettura, Università degli studi di Trieste, Trieste, Italy; 4 Newronika S.p.A., Milan, Italy; 5 U.O. Fisiopatologia,Fondazione IRCCS Ca’ Granda Ospedale Maggiore Policlinico, Milano, Italy; 6 Division of Neurology, Centre Hospitalier Universitaire de Grenoble, Grenoble, France; 7 Division of Neurosurgery, University of Toronto, Toronto, ON Canada; 8 Department of Neurology, University of Wurzburg, Würzburg, Germany

**Keywords:** Parkinson's disease, Translational research

## Abstract

This study compares the effects on motor symptoms between conventional deep brain stimulation (cDBS) and closed-loop adaptive deep brain stimulation (aDBS) in patients with Parkinson’s Disease. The aDBS stimulation is controlled by the power in the beta band (12–35 Hz) of local field potentials recorded directly by subthalamic nucleus electrodes. Eight subjects were assessed in two 8-h stimulation sessions (first day, cDBS; second day, aDBS) with regular levodopa intake and during normal daily activities. The Unified Parkinson’s Disease Rating Scale (UPDRS) part III scores, the Rush scale for dyskinesias, and the total electrical energy delivered to the tissues per second (TEEDs) were significantly lower in the aDBS session (relative UPDRS mean, cDBS: 0.46 ± 0.05, aDBS: 0.33 ± 0.04, *p* = 0.015; UPDRS part III rigidity subset mean, cDBS: 2.9143 ± 0.6551 and aDBS: 2.1429 ± 0.5010, *p* = 0.034; UPDRS part III standard deviation cDBS: 2.95, aDBS: 2.68; *p* = 0.047; Rush scale, cDBS 2.79 ± 0.39 versus aDBS 1.57 ± 0.23, *p* = 0.037; cDBS TEEDs mean: 28.75 ± 3.36 µj s^−1^, aDBS TEEDs mean: 16.47 ± 3.33, *p* = 0.032 Wilcoxon’s sign rank test). This work further supports the safety and effectiveness of aDBS stimulation compared to cDBS in a daily session, both in terms of motor performance and TEED to the patient.

## Introduction

In recent years, closed-loop systems have been used in clinical trials for the treatment of Parkinson’s Disease (PD). The power of STN beta-band oscillations (11–35 Hz) serves as a reliable neurophysiological biomarker for controlling adaptive Deep Brain Stimulation (aDBS)^1–3^. The aDBS approach is based on a simple feedback model in which biopotential recordings (namely local field potentials, LFPs) from the STN are used to estimate the clinical state of the patient and, in turn, to drive DBS parameters change^4,5^. Importantly, it was shown that the amplitude of low-beta band (13–20 Hz) is strongly correlated with motor signs severity^6,7^, thus being an ideal candidate for aDBS control. In a previous paper, we conducted a long-lasting experiment with aDBS, showing that DBS voltage linearly changing with beta power and clinical state, induces constant benefit for hours of unrestricted patient activity^8^. aDBS avoids side effects related to conventional DBS (cDBS), including gait and speech disturbances, reducing at the same time both motor and non-motor fluctuations^9,10^. Nonetheless, no study has compared to date the effects of aDBS and cDBS in a daytime window both on dyskinesia and on the three cardinal signs of PD (tremor, rigidity and bradykinesia), maintaining a regular levodopa intake, and with the patient free to move and to conduct normal activities. In this open-label, non-blinded pilot study, we aimed at investigating whether aDBS provides a better control than cDBS on the clinical motor conditions during an 8-h stimulation protocol, in conjunction of chronic levodopa assumption and without restriction on patient’s activities.

## Results

### Motor improvement between cDBS and aDBS

This experiment involved 8 subjects (see Table 1 for the details) who followed a protocol similar to the one described in Arlotti et al 2018, but undergoing one day cDBS and one day aDBS, instead of one day Stim OFF and one day aDBS (see “Methods” section). The patients here described were newly implanted DBS patients, not included in the previous experiment^8^.

**Table 1:** Patients’ experimental details.

						Preoperative response to levodopa^a^			Stimulation parameters			
Case	Sex	Age at surgery	Disease Duration [years]	Onset Side	Preoperative LEED [mg]	UPDR III score, medication “off”	UPDRS-III score, medication “on”	Beta peak [µV]	cDBS Voltage [V]	Freq.[Hz]	Pulse Width [µs]	aDBS range^b^ [V]
1	M	49	12	L	1208	41	23	0.3	2.5	130	60	0.1–2.5 (3)
2	M	64	10	L	1290	18	11	0.2	3.5	130	60	0.1–3.5 (4.2)
3	M	61	11	R	1610	26	17	2	3.5	130	60	1.0–3.5 (4.2)
4	F	51	11	L	1480	20	4	1.6	2.5	130	60	0.1–2.5 (3)
5	M	61	15	L	875	40	25	0.4	3	130	60	0.1–3.0 (3.6)
6	M	52	12	L	935	45	24	0.2	3.0	130	60	0.1–3.0 (3.6)
7^c^	M	70	19	L	1280	//	//	2.2	2.5	130	60	1.0–2.5 (3)
8	F	49	9	R	793	37	14	1.0	2.5	130	60	1.5–2.5 (3)

*LEED* levodopa equivalent daily dose, *UPDRS* Unified Parkinson’s Disease Rating Scale.

^a^Preoperative response to levodopa refers to UPDRS-III score assessed by a neurologist at the time of indication for DBS surgery (≈6 months before surgery).

^b^The first and the second value represent the real minimum and maximum stimulation voltage reached during the trial for each patient; between brackets is reported the maximum value set on the stimulator (cDBS voltage + 20%). The latter value was never reached during the trial because the aDBS was calibrated after the overnight medication washout and the maximum beta peak was never observed during the experiment.

^c^Case 7 preoperative UPDRS-III score data are missing.

Our primary outcome was motor improvement comparing aDBS with cDBS, in terms of both dyskenisias and motor scores, as assessed by UPDRS-III; the secondary outcome referred to changes in TEEDs. Baseline values (after overnight withdrawal of DBS and medication) did not differ between the two sessions (UPDRS-III, cDBS 20.86 ± 3.53 and aDBS: 20.29 ± 3.90, Rush-CDRS, cDBS: 2.50 ± 1.13 and aDBS: 1.67 ± 0.53). UPDRS-III relative score was significantly lower in the aDBS than in the cDBS session (relative UPDRS, cDBS: 0.46 ± 0.05, aDBS: 0.33 ± 0.04, *p* = 0.015, effect size *r* = 0.24, Wilcoxon’s rank sum test).

### UPDRS-III subscores analysis

**Fig. 1:**
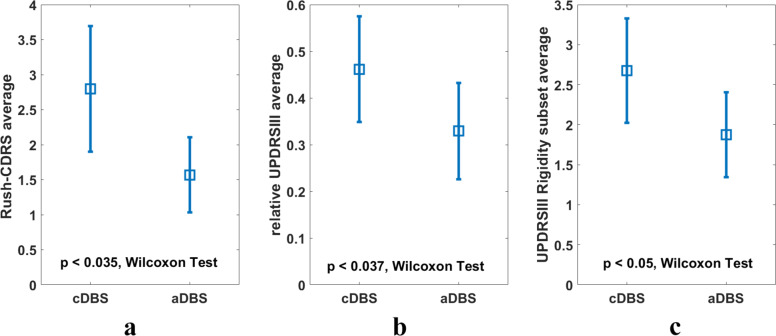
Clinical scores of the cDBS and aDBS groups. Histograms represents average and standard deviation of (**a**) Rush-DRS average, (**b**) relative UPDRS-III average, (**c**) UPDRS-III rigidity subset average.

**Fig. 2:**
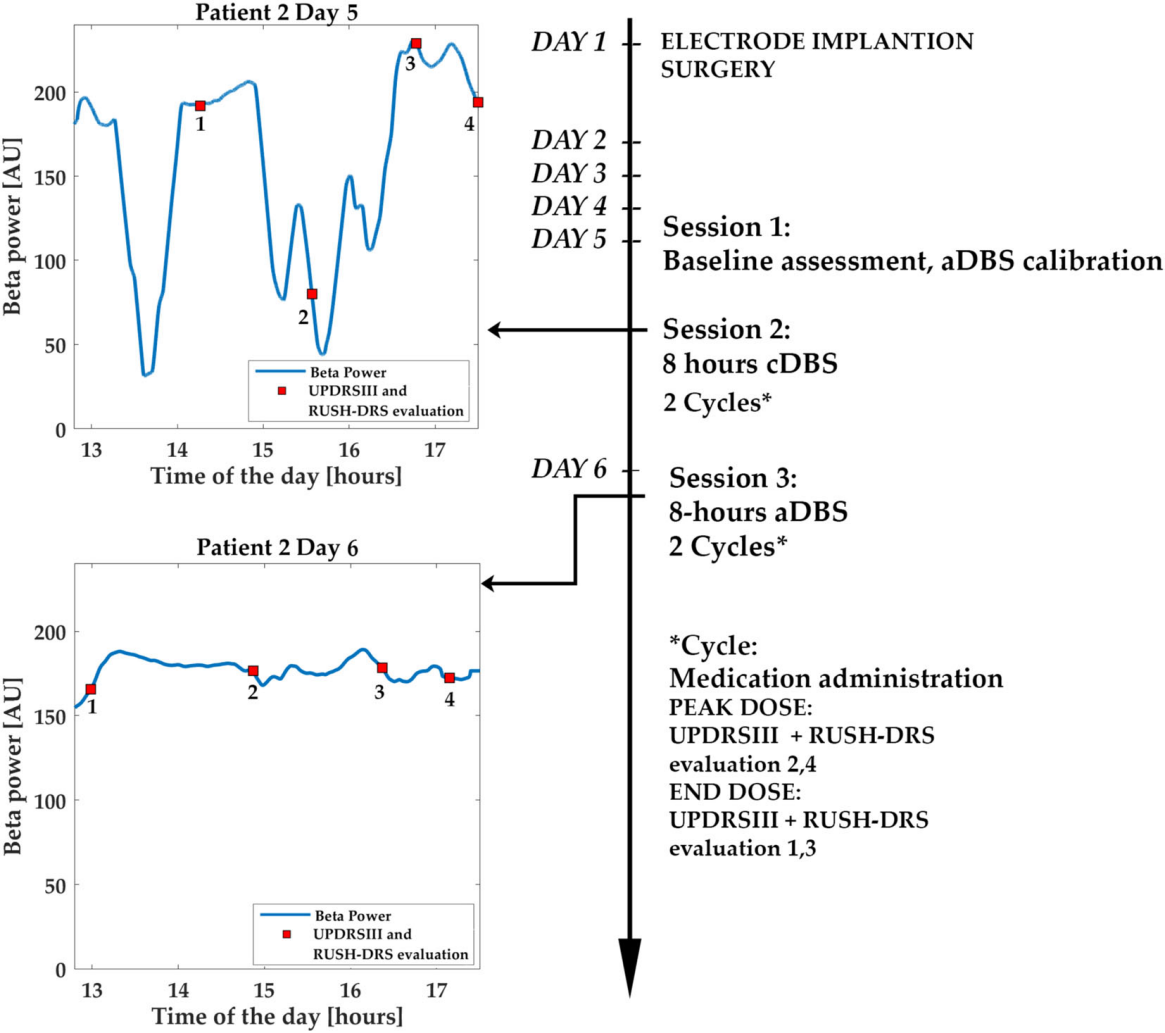
Local field potential power as recorded during the cDBS and aDBS sessions by AlphaDBS with evaluation time-points and overview of the protocol. The signal is sampled at 1 sample/s and the time format in the *x*-axis is 24-h.

Considering subscores, whereas Rest Tremor and Upper Limb Bradykinesia did not differ between aDBS and cDBS sessions, rigidity was higher during cDBS session (UPDRS-III rigidity subset mean score, cDBS: 2.9143 ± 0.6551 and aDBS: 2.1429 ± 0.5010, *p* = 0.034, effect size *r* = 0.47, Wilcoxon’s signed rank test, Fig. 1c). The reduction compared to baseline values was also calculated both for UPDRS-III and the rigidity subscore, during either cDBS or aDBS, confirming a significant improvement during adaptive stimulation (UPDRS-III: aDBS = 47.1 ± 7.5 %; cDBS = 33.2 ± 9.1%; rigidity subset: aDBS = 56 ± 6.7%; cDBS = 35.25 ± 7.9%). To evaluate the variability of the motor state during the whole session, we calculated the standard deviation of the UPDRS-III score assessed at the different time point in each experimental session. We found a significant decrease between day 1 and day 2 (UPDRS-III standard deviation day 1: 2.95, day 2: 2.68; *p* = 0.047, effect size *r* = 0.46, Wilcoxon’s signed rank test), thus implying that the UPDRS-III is less variable on the aDBS than on the cDBS session. This is in line with the stabilization of the beta band, induced by aDBS, as shown in Fig. 2.

### Levodopa-related differences

When we considered levodopa-related differences, the analysis performed between the PeakDose and EndDose values of the two sessions failed to show statistically significant difference in the total UPDRS-III, CDRS scores and in the UPDRS-III subsets between aDBS and cDBS condition. Moreover, the dyskinesia score was significantly lower in the aDBS than in the cDBS session (Rush scale, cDBS 2.79 ± 0.39 versus aDBS 1.57 ± 0.23, *p* = 0.037, effect size *r* = 0.24 Wilcoxon’s rank sum test). The Rush standard deviation scores between day 1 and day 2 are not statistically significant.

### Total electric energy delivered to the tissue

The TEEDs was calculated for 6 patients (2 patients were excluded due to a different impedance recording methodology), and it was significantly higher in the cDBS than in the aDBS session (cDBS TEEDs mean: 28.75 ± 3.36 µj s^−1^, aDBS TEEDs mean: 16.47 ± 3.33, *p* = 0.032, effect size *r* = 0.32 Wilcoxon’s sign rank test).

## Discussion

We provided the longest experiment comparing continuous with “adaptive” DBS (cDBS vs. aDBS) in freely moving patients, delivering DBS for about 8 h in an ecologic condition, with specific focus on the concurrent administration of levodopa treatment. The main results are that aDBS reduced dyskenisias, improved “rigidity” subscores and delivered a lower total energy when compared to cDBS. Equally important, the UPDRS-III scores were less variable during aDBS sessions, thus stabilizing the clinical outcome with respect to cDBS; throughout the entire experiment, no side effects were observed.

The potential benefits of aDBS approaches compared to conventional, constant-parameters DBS were already proven by different groups^3,4,9–11^. This experiment confirms and extends previous findings from our laboratory (class IV evidence), showing that aDBS is technically feasible in everyday life and provides a safe, well-tolerated, and effective treatment for the management of daily motor performance in PD patients^8^. The significant improvement of dyskinesias compared to cDBS fits in well with previous data from our laboratory, showing that when patients were under the combined effect of both levodopa and DBS (stimON/medON), aDBS was more effective on dyskinesias than conventional stimulation^12^. The second point is of key interest because no study has clearly demonstrated to date a significant improvement of rigidity compared to conventional cDBS. In particular, although a significant reduction has been also reached by cDBS, in line with data reported elsewhere^3,4,13^, aDBS improved rigidity scores to a greater extent (aDBS = 56%.0; cDBS = 35.25%). A recent study showed that aDBS, driven by brain–computer interfaces (BCI), was able to improve motor scores compared to no stimulation, cDBS and random intermittent stimulation.^3^ The authors however provided “one-shot” data, where results were collected at only one-time interval.

In another study, aDBS was compared either to cDBS and placebo stimulation in 13 PD patients, undergoing battery replacement about seven years after DBS implantation^13^. Interestingly, the authors showed that aDBS was effective in patients with bradykinetic phenotypes, delivering less stimulation than cDBS, with a more favorable speech side-effect profile. Nonetheless, they provided “one-shot” data, recorded during local anesthesia in a short time window and potentially affected by incomplete washouts among stimulation conditions. It is worth noting that the average DBS-induced improvement, either cDBS or aDBS, is lower than that reported by large DBS trials in chronic conditions, years following the implantation that report up to 70% improvement in motor scores^14,15^. This can be explained by different settings in which the measurements were taken. More specifically, the large DBS trials consider as baseline the UPDRS-III values recorded before DBS surgery (pre-operatory) and compare them with those recorded months or years after DBS turning ON, with a fully implanted system. Conversely, here we consider as baseline UPDRS-III values recorded after DBS surgery and with DBS OFF and evaluate the difference with UPDRS-III values obtained after turning DBS ON for some hours, using an externalized system connected to externalized leads. This discrepancy in the experimental setting suggests that there is the need of further clinical studies conducted in chronic patients with implanted systems to fully understand aDBS and cDBS effects and benefits.

In order to explain the improvement of rigidity induced by aDBS in comparison with cDBS, recent works have shown that different stimulation protocols induce different effects on beta oscillations, although a global reduction of beta power is provided by dopaminergic stimulation, as well as by adaptive and continuous DBS^16–18^. In particular, whereas conventional stimulation does not significantly change beta burst activity, aDBS makes dysfunctional STN networks less prone to phasic increases in beta activity^3,18,19^. Moreover, it is known that the amplitude of STN beta activity increases in proportion to burst duration, consistent with progressively increasing synchronization across a broad band from 8 to 35 Hz^20^; whereas cDBS globally suppresses beta activity, aDBS specifically shortens beta bursts duration^16,17^. Typically, bursts with shorter duration are negatively correlated with the motor impairment and rigidity of the contralateral upper limb whereas bursts with longer duration are positively correlated, especially in the “off” condition^21–23^. These observations also agree with animal studies showing that prolonged beta oscillations are pathological in parkinsonism^23^.

Overall, we cannot rule out the possibility of a “stunning” phenomenon, due to the lesional effect of the recent surgical procedure, to explain the rigidity response to cDBS, compared to the baseline and markedly outperformed with aDBS. Nonetheless, the acute setting is not particularly different from the chronic condition, as the pathological beta oscillations do not change over time, whereas changes in the electrode/tissue interface primarily affect low-frequency bands^24^. Moreover, although to a lesser extent when compared to aDBS, also cDBS markedly improved motor scores and rigidity. Finally, we also confirmed a global reduction of the TEED during aDBS in a longer period than previously described in other studies^4,12^. This TEED reduction also confirms that, even though aDBS could use the whole therapeutic window (between Vmin and Vmax), while cDBS was fixed to Vmax-20%, aDBS did not provide more stimulation than cDBS.

Our study has some limitations. First, the patient’s clinical state was evaluated by one neurologist, blinded to the stimulation protocol, without a second video referee. However, neurophysiological data, referred as to fluctuations of beta power, fit with clinical data as reported by the examiner. Second, we cannot rule out the possibility of a lesional effect, due to the surgical procedure or the peri-lesional edema, as a potential confounding factor, especially for evaluating tremor’s scores; however, the fact that both adaptive and continuous stimulation was delivered some days after the surgical procedure, allows to avoid any direct lesional effect, at least for rigidity and bradykinesia outcomes. In addition, aDBS and cDBS were administered from the same electrode contact, thus ruling out the possibility of a differential effect due to stimulation position. Third, aDBS and cDBS were not administered in random order, because the data from the first day were necessary to check the calibration parameters for aDBS. However, a full overnight DBS withdrawal between Day 1 and Day 2 could be enough to minimize possible carry-over effects. In addition, patients were free to move and to perform their normal activities during the day, thus minimizing the stress related to the experimental procedure, and thus ruling out the possibility of a bias due to the patient feeling more relaxed during the second experimental session than during the first.

Finally, another limitation may be the anatomical variability of the target, as probably the dorsolateral STN was not stimulated in all patients given the depth of the stimulating electrodes placed in some cases nearby the caudal STN border zone (see Table 2). Nonetheless, converging evidence suggest that beta oscillations are also locally generated in the ventral STN and in the substantia nigra *pars reticulata* (SNr)^25,26^ and may be correlated to the development of limbs’ akinesia, as described in animal^27^.

**Table 2:** Target coordinates of the contact stimulated.

	Recording contact pair			Left target [mm]			Right target [mm]		
Case	Right	Left	ACPC [mm]	Lateral	Posterior	Inferior	Lateral	Posterior	Inferior
1	0–3	8–11	26.12	10.93	1.43	6.26	10.24	1.25	7
2	0–3	8–11	26.31	9.45	2.89	8.38	11.23	2.39	5.38
3	0–3	8–10	24.69	11.19	2.96	4.43	8.88	0.57	4.43
4	0–2	8–11	24.81	11.37	1.89	2.67	10.82	2.05	4.83
5	0–2	8–10	27.03	12.48	1.43	5.09	10.16	0.33	5.64
6	0–3	8–11	22.61	10.21	4.51	6.61	8.94	5.43	7.9
7	0–2	8–11	24.65	10.48	2.08	8.5	10.54	1.54	7.78
8	0–3	9–11	25.29	9.95	1.23	4.59	9.68	3.24	4.86

Patients with tremor-dominant disease, may benefit from additional biomarkers than beta oscillations and lower threshold to trigger stimulation than their rigid-bradykinetic counterparts^7,13,28,29^. In conclusion, our work further confirms the safety and effectiveness of aDBS, as compared to cDBS, both in terms of motor performances and of DBS side effects, in a day-long time window. Further studies and larger clinical trials comparing aDBS and cDBS are needed to confirm our results, especially in the long-term period, in freely moving parkinsonian patients.

## Methods

To study the benefits of aDBS versus the cDBS we enrolled eight patients with advanced PD who underwent surgery for STN-DBS electrode implantation at the Neurosurgery Unit of Fondazione IRCCS Ca’ Granda Ospedale Maggiore Policlinico in Milan, Italy, from July 2017 to October 2018. This patients’ series followed another patients’ series reported in Arlotti 2018^8^, with no overlap. Patients signed written Informed Consent to participate to the study which was approved by the local Ethical Committee of the Fondazione IRCCS Ca’ Granda Ospedale Maggiore Policlinico, and conformed to the Declaration of Helsinki. The experiment is conducted using the *AlphaDBS*_ext device^5,8^. Patients’ experimental details are reported in Table 1. In summary, AlphaDBS_ext is a CE-marked external and portable device allowing the synchronous recording of LFPs from DBS electrodes and cDBS/aDBS delivery. AlphaDBS_ext is a unique device fully integrating real-time closed-loop aDBS capability, without the need of external or asynchronous processing.

### LFP recording and DBS administration

LFPs were recorded from two contacts available on the Medtronic 3389 DBS electrode (Medtronic, Inc), both when DBS is turned ON or OFF. In cDBS mode, the device provided stimulation through one of the contacts not used for recording (according to clinician’s choice) using a fixed-parameters set (amplitude, frequency, and pulse width set by the neurologist). In aDBS mode, the device delivered stimulation in which the amplitude was changed in real time according to the analysis of the recorded beta band LFPs. The voltage varied linearly with the beta power, within a specified therapeutic window (Vmin-Vmax) chosen by the neurologist (see next section for details).

### Experimental procedure

The experimental procedure was similar to the one described in Arlotti et al, 2018^8^, but instead of testing aDBS vs StimOFF, was designed to test aDBS vs cDBS. The experiment took place five days after the electrode implantation surgery, and consisted of three sections lasting in total 2 days (Fig. 2):

- Session 1: baseline assessment and aDBS calibration (day 5);
- Session 2: 8 h cDBS (day 5);
- Session 3: 8-h aDBS (day 6).

Session 1. aDBS calibration. After overnight PD medication withdrawal, a baseline assessment including the Unified Parkinson’s Disease Rating Scale (UPDRS)-III, the Rush filming protocol and the Dyskinesias Rating Scale (RUSH - CDRS) REF was performed by an experienced physician. Then, the calibration phase comprised three steps: (a) electrode contacts selection: all the contacts combinations were probed and recorded to select the pair with a prominent non-artefactual beta peak, among those not including the stimulation contact as selected from the intraoperative data. (b) Therapeutic window: the stimulation contact was selected based on the best clinical outcome on rigidity subscores, as assessed in the operative room (see Table 2 for target coordinates); an experienced neurologist assessed the therapeutic window that was needed to define the lowest and highest amplitudes to be used by aDBS. More specifically, the minimum value (Vmin) was defined as the minimum voltage providing a benefit to the patient, namely at least 20% improvement in rigidity; and the maximum value (Vmax) as the maximum voltage before the occurrence of side effects. aDBS amplitude was therefore set to change its value in the Vmin–Vmax window, according to the change in the amplitude of the beta band (linear adaptation^5^). This therapeutic window (Vmin-Vmax) prevented stimulation to be switched off when beta was very low, in order to avoid transition effects due to switching DBS on and off and also allowed aDBS to reach the maximum amplitude possible (threshold for side effects, Vmax), when beta is very high, considering that this amplitude would be applied only for short time windows (until the beta decreases). cDBS amplitude, on the other hand, was set to a fixed value, calculated as Vmax (threshold for side effects)-20%, thus guaranteeing the trade-off between effective stimulation and avoided side effects. Frequency and pulse width remained constant for every patient, and are set to 130 Hz and 60 µs respectively. c) Beta peak selection: the frequency of the beta peak (frequency with the maximum beta power) is selected as the center of a personalized beta band (maximum frequency ± 2 Hz) on the contact pair chosen in step a) and set as biomarker for aDBS.

Session 2. Eight-hours cDBS. After baseline assessment and calibration, cDBS was turned on and the first L-dopa administration was given. cDBS is delivered for 8 h with an amplitude of Vmax -20% (see point a and Table 1). Meanwhile, the regular intake of L-dopa was resumed during the session. UPDRS-III and DRS evaluations are performed by an experienced neurologist during the peak (PeakDose) and the end (EndDose) of L-Dopa efficacy. At the end of the session, cDBS is turned OFF.

Session 3. Eight-hours aDBS. On the second day (day 6 after the implantation surgery), after overnight medication withdrawal, aDBS was turned ON for 8 h using the parameters chosen in calibration (personalized beta band and therapeutic window, see point a and Table 1). Then, the experimental session was repeated as the day before.

### Data analysis

The UPDRS-III and DRS data were collected for all the patients and categorized in “cDBS” (day 5) and “aDBS” (day 6), and of every day the subsets of “PeakDose” and “EndDose” categories were created. UPDRS-III items were also divided into three different subsets: Rest Tremor (items 3a to 3e), Rigidity (items 5a to 5e), and Upper Limb Bradykinesia (items 6a to 8b). Another group of subsets was created using the PeakDose and EndDose values considering the total scores to analyze the motor status of the patient in two different pharmacological phases between the two sessions. To take into account the high variability between patients in the total UPDRS-III score, due to heterogeneity of symptoms and the limited number of samples, a normalization was performed, according to the formula

1 $${\rm{relativeUPDRS}} = \frac{{{{{\rm{UPDRS}}}}_{{{{\rm{SCORE}}}}} - {{{\mathbf{min}}}}\left( {{{{\rm{UPDRS}}}}_{{{{\rm{SCORE}}}}}} \right)}}{{\max \left( {{{{\rm{UPDRS}}}}_{{{{\rm{SCORE}}}}}} \right) - {{{\mathbf{min}}}}\left( {{{{\rm{UPDRS}}}}_{{{{{{\rm{SCORE}}}}}}}} \right)}}$$

where relativeUPDRS: the relative UPDRS-III total score; UPDRS_score_: the absolute UPDRS-III total score of a single patient; max() and min(): absolute maximum and minimum UPDRS-III score of a single patient (considering both sessions). Therefore, to obtain the relativeUPDRS, the raw UPDRS-III was normalized by the maximum change recorded in each patient. This allows to compare patients having different UPDRS-III ranges in different conditions (e.g., a score of 2 in a patient having a range between 1 and 10 is different from a score of 2 in a patient having a range between 2 and 40). This relative score was applied in order to take into account the variation of UPDRS-III during the sessions, excluding the interpatient expected variability. Conversely, the three UPDRS-III subsets (Rest Tremor, Rigidity and Upper Limb Bradykinesia) and the DRS total score were analyzed using the absolute subset score (without normalization) because of the low score range due to the limited number of items. We also analyzed the mean total electrical energy delivered to the tissues per second (TEEDs), using the formula by Koss et al.^30^ in the two daily sessions.

The statistical analysis was carried out using the software MATLAB Version 9.3.0.713579 (R2017b, The MathWorks Inc.,USA). All data are expressed as [mean ± standard error]. Due to the small sample size all the statistical tests were non-parametric (two tailed Wilcoxon’s rank sum test and two tailed paired signed rank tests); effect size r is estimated for non-parametric tests as reported in Fritz et al.^31^.

### Reporting summary

Further information on research design is available in the Nature Research Reporting Summary linked to this article.

## Supplementary information

Reporting Summary

## Data Availability

The authors confirm that the data supporting the findings of this study are available upon request.

## Code availability

The authors confirm that code is available upon request from the corresponding author.
