# Author Correction: Eight-hours conventional versus adaptive deep brain stimulation of the subthalamic nucleus in Parkinson’s disease

**DOI:** 10.1038/s41531-022-00281-3

**Published:** 2022-02-15

**Authors:** Tommaso Bocci, Marco Prenassi, Mattia Arlotti, Filippo Maria Cogiamanian, Linda Borellini, Elena Moro, Andres M. Lozano, Jens Volkmann, Sergio Barbieri, Alberto Priori, Sara Marceglia

**Affiliations:** 1 “Aldo Ravelli” Research Center for Neurotechnology and Experimental Brain Therapeutics, Department of Health Sciences, University of Milan Medical School, Milan, Italy; 2 III Neurology Clinic, ASST Santi Paolo e Carlo, Milan, Italy; 3 Dipartimento in Ingegneria e Architettura, Università degli studi di Trieste, Trieste, Italy; 4 Newronika S.p.A., Milan, Italy; 5 U.O. Fisiopatologia,Fondazione IRCCS Ca’ Granda Ospedale Maggiore Policlinico, Milano, Italy; 6 Division of Neurology, Centre Hospitalier Universitaire de Grenoble, Grenoble, France; 7 Division of Neurosurgery, University of Toronto, Toronto, ON Canada; 8 Department of Neurology, University of Wurzburg, Würzburg, Germany

**Keywords:** Parkinson's disease, Translational research

Correction to: *npj Parkinson’s Disease* 10.1038/s41531-021-00229-z, published online 28 September 2021

“In this article the author name Linda Borellini was incorrectly written as Linda Borrellini. The original article has been corrected.”

