# Supplementary material for: Eight-hours conventional versus adaptive deep brain stimulation of the subthalamic nucleus in Parkinson’s disease
Source: NPJ Parkinsons Dis. 2021 Sep 28;7:88. doi: 10.1038/s41531-021-00229-z (PMC8478873; doi:10.1038/s41531-021-00229-z)
Supplement: Supplementary file 1 — Reporting Summary [file 41531_2021_229_MOESM1_ESM.pdf]

nature research

Corresponding author(s): Professor Alberto Priori
alberto.priori@unimi.it

Last updated by author(s): Dec 9, 2020

Reporting Summary

Nature Research wishes to improve the reproducibility of the work that we publish. This form provides structure for consistency and transparency in reporting. For further information on Nature Research policies, see our Editorial Policies and the Editorial Policy Checklist.

Statistics

For all statistical analyses, confirm that the following items are present in the figure legend, table legend, main text, or Methods section.

n/a	Confirmed	
☐	☒	The exact sample size (n) for each experimental group/condition, given as a discrete number and unit of measurement
☐	☒	A statement on whether measurements were taken from distinct samples or whether the same sample was measured repeatedly
☐	☒	The statistical test(s) used AND whether they are one- or two-sided<br>*Only common tests should be described solely by name; describe more complex techniques in the Methods section.*
☐	☒	A description of all covariates tested
☐	☒	A description of any assumptions or corrections, such as tests of normality and adjustment for multiple comparisons
☐	☒	A full description of the statistical parameters including central tendency (e.g. means) or other basic estimates (e.g. regression coefficient) AND variation (e.g. standard deviation) or associated estimates of uncertainty (e.g. confidence intervals)
☐	☒	For null hypothesis testing, the test statistic (e.g. F, t, r) with confidence intervals, effect sizes, degrees of freedom and P value noted<br>*Give P values as exact values whenever suitable.*
☒	☐	For Bayesian analysis, information on the choice of priors and Markov chain Monte Carlo settings
☒	☐	For hierarchical and complex designs, identification of the appropriate level for tests and full reporting of outcomes
☒	☐	Estimates of effect sizes (e.g. Cohen's d, Pearson's r), indicating how they were calculated

Our web collection on statistics for biologists contains articles on many of the points above.

Software and code

Policy information about availability of computer code

Data collection	The data collection was implemented with MATLAB Version 9.3.0.713579 (R2017b, The MathWorks Inc., USA).
Data analysis	The statistical analysis was carried out using the software MATLAB Version 9.3.0.713579 (R2017b, The MathWorks Inc., USA). The authors confirm that code is available upon request from the corresponding author.

For manuscripts utilizing custom algorithms or software that are central to the research but not yet described in published literature, software must be made available to editors and reviewers. We strongly encourage code deposition in a community repository (e.g. GitHub). See the Nature Research guidelines for submitting code & software for further information.

Data

Policy information about availability of data

All manuscripts must include a data availability statement. This statement should provide the following information, where applicable:

- Accession codes, unique identifiers, or web links for publicly available datasets
- A list of figures that have associated raw data
- A description of any restrictions on data availability

The authors confirm that the data supporting the findings of this study are available upon request.

April 2020

Field-specific reporting

Please select the one below that is the best fit for your research. If you are not sure, read the appropriate sections before making your selection.

☒ Life sciences ☐ Behavioural & social sciences ☐ Ecological, evolutionary & environmental sciences

For a reference copy of the document with all sections, see nature.com/documents/nr-reporting-summary-flat.pdf

Life sciences study design

All studies must disclose on these points even when the disclosure is negative.

Sample size	This was a pilot study, not intended to provide superiority claims. The number of patients in the study is in line with previous published papers on the same topic.
Data exclusions	No data were excluded from the analysis
Replication	The study design is similar to the one published in Arlotti et al 2018. In addition, the main findings are in agreement with the literature on aDBS
Randomization	In this experimental design, as explained in the manuscript, it was not possible to randomize between the two conditions (cDBS, aDBS) because the data recorded during the application of cDBS were needed to confirm the calibration of aDBS. This limitation is also discussed in the manuscript.
Blinding	Because it was not possible to randomize, blinding was not applicable.

Reporting for specific materials, systems and methods

We require information from authors about some types of materials, experimental systems and methods used in many studies. Here, indicate whether each material, system or method listed is relevant to your study. If you are not sure if a list item applies to your research, read the appropriate section before selecting a response.

Materials & experimental systems

n/a	Involved in the study	
☒	☐	Antibodies
☒	☐	Eukaryotic cell lines
☒	☐	Palaeontology and archaeology
☒	☐	Animals and other organisms
☐	☒	Human research participants
☒	☐	Clinical data
☒	☐	Dual use research of concern

Methods

n/a	Involved in the study	
☒	☐	ChIP-seq
☒	☐	Flow cytometry
☒	☐	MRI-based neuroimaging

Human research participants

Policy information about studies involving human research participants

Population characteristics	Patients with Parkinson's disease, predominantly rigid-akinetic, indicated for deep brain stimulation implant and undergoing the stereotactic neurosurgical procedures for DBS electrode implant.
Recruitment	All patients undergoing DBS implant at the Neurosurgery Unit of Fondazione IRCCS Ca' Granda Ospedale Maggiore Policlinico in Milan, Italy, not presenting post-surgical complications, were asked for consent to participate to the study
Ethics oversight	The study was approved by the local Ethical Committee of the Fondazione IRCCS Ca' Granda Ospedale Maggiore Policlinico.

Note that full information on the approval of the study protocol must also be provided in the manuscript.
